# Genome sequence of the lytic bacteriophage Alucard, a cluster EE actinophage

**DOI:** 10.1128/mra.01017-23

**Published:** 2023-12-05

**Authors:** Samuel R. Cousins, Gemma R. Dufour, Kendra Law, Robert M. Nichols, Bradi J. Sladek, Christopher R. Aniapam, Brian P. Tarbox, Emily F. Savage

**Affiliations:** 1 Biology, Southern Maine Community College, South Portland, Maine, USA; Portland State University, Portland, Oregon, USA

**Keywords:** bacteriophage, SEA-PHAGES, genome, bioinformatics, actinobacteriophages, sequencing

## Abstract

Bacteriophage Alucard is a lytic phage isolated from the soil collected in southern Maine on *Microbacterium foliorum* NRRL B-24224. Alucard has siphovirus morphology with a 17,363-bp genome encoding 25 putative genes. Based on gene content similarity to actinobacteriophages, Alucard is assigned to cluster EE.

## ANNOUNCEMENT

*Actinobacteria* phages are increasingly being developed as therapeutics for treating bacterial infections (1). To shed more light on the ecology and diversity of actinobacteriophages, here we report on bacteriophage Alucard, isolated using *Microbacterium foliorum* (NRRL B-24224), that contains no detectable prophages or CRISPR systems and is therefore ideal as an isolation host (2).

**Fig 1:**
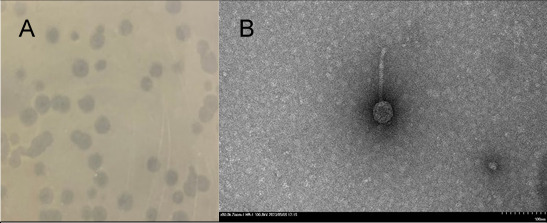
(**A**) Three-millimeter (*n* = 5) diameter plaques formed on *M. foliorum* NRRL B-24224 by Alucard after 48-h incubation at 30°C on PYCa media. (**B**) Virion of phage Alucard by TEM (Hitachi HT7800, 120 kV, accelerating voltage 100 kV) using negative stain (1% uranyl acetate). Alucard has a siphovirus morphology with a tail of 103 nm and an isometric capsid of 44 nm in diameter (*n* = 1).

Bacteriophage Alucard was isolated from moist and dark soil collected on a tomato patch in a community garden at Southern Maine Community College (Global Positioning System 43.647224 N, 70.227765 W) using standard procedures (3). A 5 g sample of soil was added to PYCa (peptone, yeast extract, and calcium chloride) liquid media and inoculated with *M. foliorum*. After 4 days of incubation at 30°C, the sample was filtered (0.22 µm filter), and the filtrate was spotted onto PYCa top agar pre-mixed with *M. foliorum*, yielding phage Alucard after 48 h at 30°C. Alucard, which formed clear plaques with defined borders and an average diameter of 3 mm (*n* = 5), was purified through three rounds of plating (Fig. 1A). Negative stain transmission electron microscopy revealed the Alucard to have siphovirus morphology (Fig. 1B).

Alucard DNA was extracted from a lysate using the Promega Wizard DNA clean up kit. A sequencing library was prepared using the NEB Ultra II Library Kit, followed by sequencing conducted on the Illumina MiSeq sequencer using v3 reagents, yielding 604,392 150-base single-end reads, providing 5,143-fold coverage. Raw reads were assembled and checked by Newbler version 2.9 (4) and Consed V29 (5) using default parameters.

The genome sequence was annotated using DNA Master (V5.23.6, Build 2705 24 Oct 2021). Glimmer (v3.02b) (6) and GeneMark (PS-v1.2) (7) were used to detect protein-coding genes. Starterator (v3.02) (8), Phamerator (Actino_Draft V509) (9), BLASTp (actinobacteriophage proteins, non-redundant protein sequences) (10), and HHPRED (PDB_mmCIF70, SCOPe70, Pfam-A, NCBI_Concerved_Domains) (11) were used to call 25 putative genes and assign putative functions to 18. No tRNAs were identified using Aragorn (v1.2.41.c.) (12) and DeepTMHMM (v1.2.33.c.) (13), and SOSUI (v1.11) (14) identified two membrane proteins. All software was used with the default settings.

Alucard was assigned to phage EE cluster based on gene content similarity (GCS) of 35% or higher to phages in the Actinobacteriophage database (https://phagesdb.org/), using the phagesDB GCS tool (15, 16). Alucard adds to the known gene congruency of cluster EE phages. Cluster EE phages feature uncharacteristically small genomes, interesting gene fusions such as the capsid maturation protease, scaffolding, and HK97-like capsid subunit into one gene (17), and a conserved translational frameshift to form the tail assembly chaperone fusion protein. Alucard contains the typical lysis cassette for this cluster, containing *lsr2*, HNH nuclease, and an endolysin (17, 18).

## Data Availability

Sequencing results for Alucard are available in GenBank with accession no. OR521072 and Sequence Read Archive (SRA) accession no. SRX20165759.
